# Characterization of Membrane Topology and Retention Signal of Pestiviral Glycoprotein E1

**DOI:** 10.1128/JVI.00521-21

**Published:** 2021-07-12

**Authors:** Yu Mu, Christina Radtke, Birke Andrea Tews, Gregor Meyers

**Affiliations:** a Institut für Immunologie, Friedrich-Loeffler-Institut, Greifswald-Insel Riems, Germany; b Institut für Infektionsmedizin, Friedrich-Loeffler-Institut, Greifswald-Insel Riems, Germany; Instituto de Biotecnologia/UNAM

**Keywords:** envelope protein, glycoprotein surface expression, intracellular retention signal, membrane topology, pestivirus, viral polyprotein processing

## Abstract

Pestiviruses are members of the family *Flaviviridae*, a group of enveloped viruses that bud at intracellular membranes. Pestivirus particles contain three glycosylated envelope proteins, E^rns^, E1, and E2. Among them, E1 is the least characterized concerning both biochemical features and function. E1 from bovine viral diarrhea virus (BVDV) strain CP7 was analyzed with regard to its intracellular localization and membrane topology. Here, it is shown that even in the absence of other viral proteins, E1 is not secreted or expressed at the cell surface but localizes predominantly in the endoplasmic reticulum (ER). Using engineered chimeric transmembrane domains with sequences from E1 and vesicular stomatitis virus G protein, the E1 ER-retention signal could be narrowed down to six fully conserved polar residues in the middle part of the transmembrane domain of E1. Retention was observed even when several of these polar residues were exchanged for alanine. Mutations with a strong impact on E1 retention prevented recovery of infectious viruses when tested in the viral context. Analysis of the membrane topology of E1 before and after the signal peptide cleavage via a selective permeabilization and an *in vivo* labeling approach revealed that mature E1 is a typical type I transmembrane protein with a single span transmembrane anchor at its C terminus, whereas it adopts a hairpin-like structure with the C terminus located in the ER lumen when the precleavage situation is mimicked by blocking the cleavage site between E1 and E2.

**IMPORTANCE** The shortage of specific antibodies against E1, making detection and further analysis of E1 difficult, resulted in a lack of knowledge on E1 compared to E^rns^ and E2 with regard to biosynthesis, structure, and function. It is known that pestiviruses bud intracellularly. Here, we show that E1 contains its own ER retention signal: six fully conserved polar residues in the middle part of the transmembrane domain are shown to be the determinants for ER retention of E1. Moreover, those six polar residues could serve as a functional group that intensely affect the generation of infectious viral particles. In addition, the membrane topology of E1 has been determined. In this context, we also identified dynamic changes in membrane topology of E1 with the carboxy terminus located on the luminal side of the ER in the precleavage state and relocation of this sequence upon signal peptidase cleavage. Our work provides the first systematic analysis of the pestiviral E1 protein with regard to its biochemical and functional characteristics.

## INTRODUCTION

The genus *Pestivirus* belongs to the family *Flaviviridae*. Originally, four recognized species have been classified into the genus *Pestivirus*—bovine viral diarrhea virus type 1 (BVDV-1), BVDV-2, classical swine fever virus, and border disease virus of sheep—but many new isolates have recently been found (see references 1, 2, and 3 for a review). In the latest online report (10th) of the ICTV, the genus *Pestivirus* has been subdivided into 11 different species correspondingly (4). Members of the genus *Pestivirus* have been found in a broad variety of farm or wild animals, with a clear focus on pigs and ruminants. In these hosts, the viruses induce diverse clinical manifestations and cause very severe financial losses in the livestock farming industry (3).

Pestivirus virions are enveloped and contain four structural proteins, including one basic core protein C and three envelope (E) glycoproteins (E^rns^, E1, and E2). E^rns^ and E2 have been shown to be accessible on the viral particles (5–8). Cellular signal peptidase (SP) is responsible for the cleavage at the C/E^rns^ site, followed by further processing of the remaining capsid protein bound C-terminal signal sequence by signal peptide peptidase (9, 10). SP is also responsible for processing at the E^rns^/E1, E1/E2, E2/p7, and p7/NS2 sites (10–13).

The shortage of robustly reacting specific antibodies against E1 hampered the detailed analysis of E1, resulting in a lack of knowledge on E1 compared to E^rns^ and E2 with regard to biosynthesis, structure, and function. Biochemical data on E1 have mainly been obtained as by-products during analyses focusing on combinations with the other two envelope proteins, namely, the rather stable E^rns^-E1 precursor generated during processing and the covalently linked E1-E2 heterodimer found in infected cells and virions that is crucial for infection of target cells (5, 10). Immunoprecipitation of the heterodimer with antibodies against E2 and subsequent polyacrylamide gel electrophoresis (PAGE) under reducing conditions allowed first demonstration of E1 and determination of its apparent molecular weight (7).

Pestiviruses have long been considered to bud intracellularly, and ultrastructure analyses conducted of cells infected with the pestivirus Giraffe-1 confirmed this conclusion (14). Therefore, the envelope proteins of pestiviruses have to accumulate in a specific intracellular compartment to allow virus assembly and budding at the compartment membrane. The glycoprotein E1/E2 complexes of HCV were shown to accumulate in the endoplasmic reticulum (ER) where virus budding takes place (15). Also, pestiviral E^rns^ and E2 were shown to be concentrated in the ER and appropriate retention signals have been identified (16–18). The intracellular location of E1 is not known. The formation of E1/E2 heterodimers strongly indicates that E1 should locate in the ER or a compartment close to the ER, but this has not been proven thus far; if so, it is unclear whether E1 contains an ER localization signal of its own or relies on the signal provided by the E2 moiety of the heterodimer. The fact that E1 is able to rescue intracellular retention for some E2 retention defective mutants gave the first hint that E1 must have its own retention signal (17).

In contrast to E^rns^ and E2, data on the structure or membrane topology of E1 have not been published. Because of the similarity to HCV and primary sequence characteristics it has been hypothesized that E1 represents a type I transmembrane protein with a C-terminal membrane anchor. Recently, published data suggested that E1 contains one transmembrane helix with two amphipathic perimembrane helices located upstream of the transmembrane (TM) helix (19). However, the latter publication is based only on computational modeling tools used to simulate and predict the secondary structure of pestivirus E1 and E2. Thus, the E1 membrane topology awaits detailed experimental analysis.

In this study, we analyzed the intracellular localization of E1 and identified the determinants for this localization. In addition, we also elucidated its membrane topology in the pre- and postcleavage states.

## RESULTS

### Subcellular localization of pestivirus E1.

We wanted to analyze the subcellular location of E1, especially when expressed in the absence of other viral proteins. Since no antibodies for robust and specific detection of BVDV E1 were available, we established a cDNA construct for transient expression of E1 with an N-terminal hemagglutinin (HA) tag (construct pYM-13), to be able to detect E1 with HA-tag-specific antibodies.

**FIG 1:**
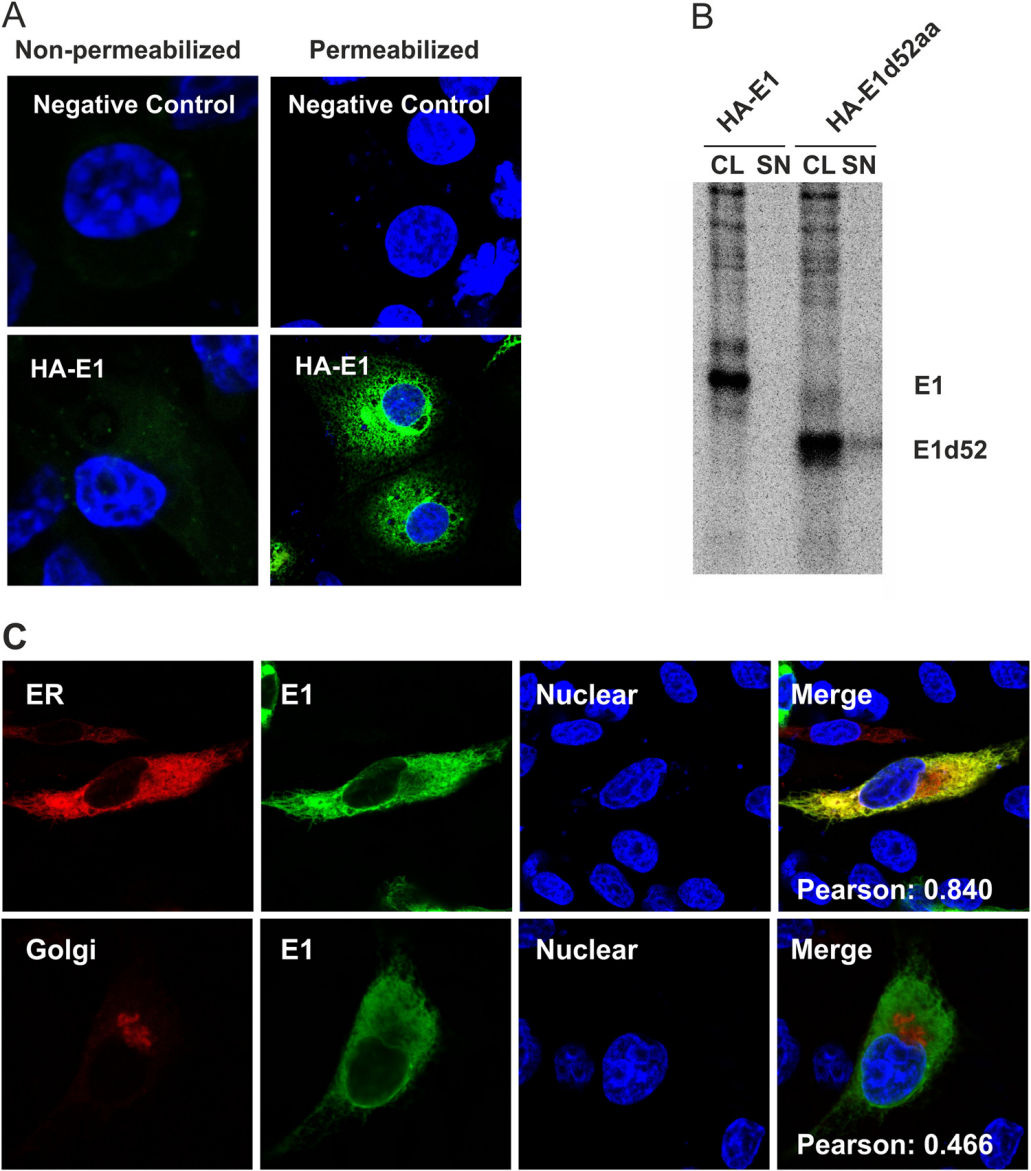
Subcellular localization of E1. (A) RK-13 cells were transfected in duplicate with the pCI empty vector or HA-tagged E1 expression plasmid (pYM-13). At 24 h posttransfection, the cells were analyzed by indirect immunofluorescence. The RK-13 cells were fixed with 4% PFA, and then one sample was permeabilized with 0.05% Triton X-100, whereas the other sample was left without detergent. Immunostaining was done with anti-HA MAb and secondary Alexa Fluor 488-anti-mouse antibody. Nuclei were stained with DAPI. (B) Immunoprecipitation for testing the secretion of E1. The expression plasmid (pYM-13) coding for HA-tagged E1 was expressed in BHK-21 cells using the vaccinia virus MVA T7 expression system. As a control, a version of the plasmid coding for an E1 protein with a carboxy-terminal deletion of 52 aa was transfected in parallel as a control. Expressed proteins were labeled with ^35^S-labeled amino acids. From the supernatant (SN) and cell lysate (CL) of the transfected cells, proteins reacting with a specific antiserum directed against the HA tag were precipitated. The samples were treated with PNGase F and then separated by SDS-PAGE under reducing conditions. The labeled proteins were detected on imaging plates. Unfortunately, the anti-HA sera that we tested all reacted only weakly in precipitation experiments, so that long exposure times resulting in a quite high background were needed. (C) For E1 intracellular localization analysis, BHK-21 cells were cotransfected with the HA-tagged E1 expression plasmid and pDsRed-ER or pDsRed-Golgi, respectively. At 24 h posttransfection, the cells were fixed by 4% PFA, permeabilized with 0.05% Triton X-100, and stained with specific antibodies against HA (green). Compartments (ER or Golgi) are indicated in red. Nuclei were stained with DAPI. Analysis was done with a Leica SP5 confocal laser scan microscope.

As a first test, we transfected RK-13 cells with plasmid pYM-13 and looked for transient expression via immunofluorescence. Specific staining was observed when the cells were permeabilized with 0.05% Triton X-100 showing that the tagged E1 could be identified with the HA-tag antibodies (Fig. 1A). To check whether E1 stays within the cell or is transported to the cell surface, we conducted the same experiment without permeabilization. As shown in Fig. 1A, there was no HA signal under both nonpermeabilized and permeabilized condition in the negative-control samples (RK-13 cells transfected with pCI empty vector), indicating good specificity of the HA-tag detection. Importantly, there was also no specific signal on the cell surface under nonpermeabilized condition in E1 transfected cells, in contrast to the strong signal within the cells under permeabilized condition.

We also checked that there was no detectable secretion of E1 in the supernatant of transfected cells via metabolic labeling and immunoprecipitation from cell lysates and cell culture supernatants (Fig. 1B). These results indicated that E1 is retained within the cell, even when expressed in the absence of other viral proteins, whereas a version with a deletion of the mainly hydrophobic carboxy-terminal 52 amino acids (aa) of E1 was clearly detectable in the supernatant, proving that secreted E1 could be detected with the employed method.

To investigate the subcellular localization of E1 in different cell organelles, cells were cotransfected with pYM-13 (HA-E1) and the marker plasmids pDsRed-ER or pDsRed-Golgi to label the ER or Golgi apparatus, respectively. The results were analyzed with a confocal fluorescence microscope (Fig. 1C). Comparison of the E1 signal to that of the compartment markers revealed that E1 was mainly concentrated in the ER (Pearson’s correlation coefficient [PCC] of 0.840), while no detectable localization in the Golgi compartment was observed (PCC of 0.466). These results demonstrated that E1 has to contain an ER retention signal of its own instead of being localized at the site of virus budding via the interaction with other viral proteins (e.g. E2).

### The transmembrane anchor of E1 is a determinant for ER retention.

**FIG 2:**
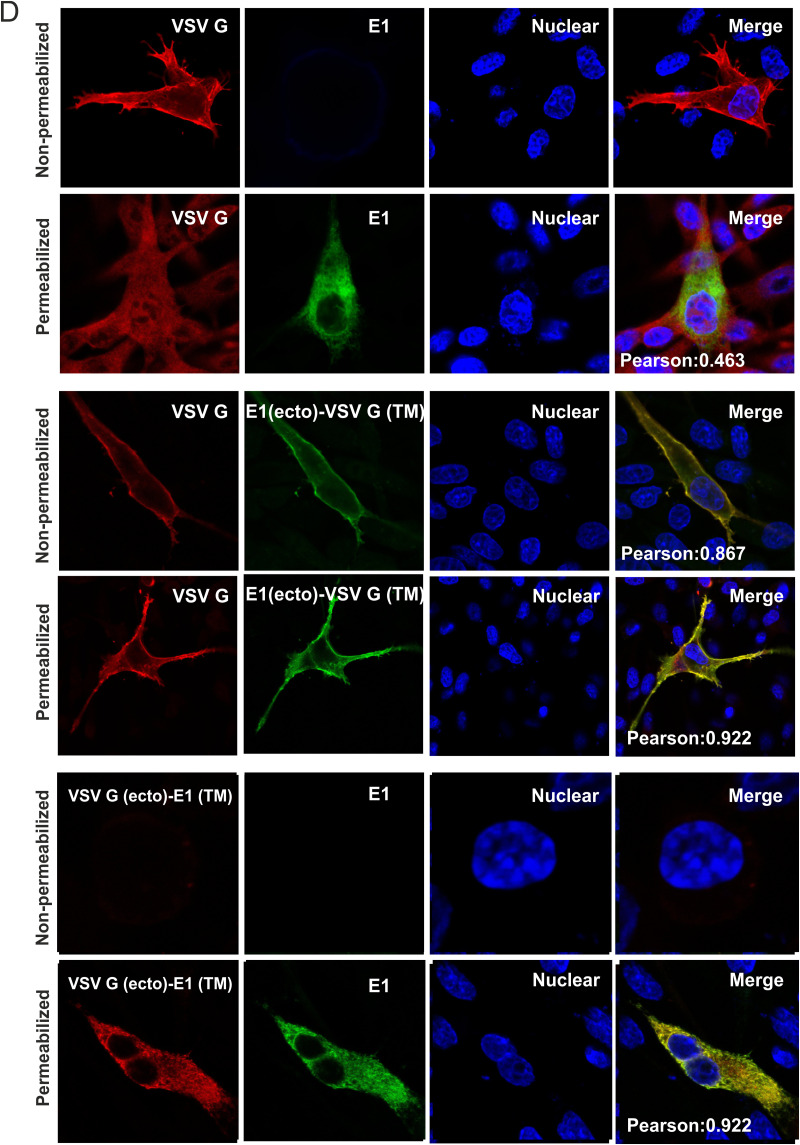
(Continued).

It was shown that the E1 protein of hepatitis C virus (HCV) contains an intrinsic retention signal within its transmembrane domain (TMD). For both E^rns^ and E2 of pestiviruses, the C-terminal membrane anchors were found to be responsible for their retention (16, 17, 20). In order to investigate whether the proposed TMD of E1 plays a similar role, fusion proteins composed of parts of a protein naturally exported to the cell surface and parts of E1 were constructed (as shown in Fig. 2A). A commonly used partner protein for such analyses is vesicular stomatitis virus G protein (VSV-G). VSV-G is a typical type I transmembrane protein that is normally expressed on the plasma membrane of the cells. Based on preliminary data in our lab, the putative TMD of E1 should encompass the last ca. 30 aa at the C terminus (ca. aa 166 to 195), while the rest is supposed to represent the ectodomain of E1. It is known that the TMD of VSV-G is a short peptide that extends from I465 to C489 (aa sequence IASFFFIIGLIIGLFLVLRVGIHLC).

Cells transfected with plasmid p838 expressing full-length VSV-G were used as a control of cell surface expression, since there is no retention signal within VSV-G. To confirm or refute the hypothesis that the TMD of E1 is responsible for E1 retention, we generated two additional cDNA constructs that code for chimeric proteins containing either the VSV-G ectodomain (aa 1 to 464) with the E1 TMD (aa 166 to 195; pYM-57) or the E1 ectodomain (aa 1 to 165) with the VSV-G TMD (aa 465 to 489; pYM-56). Cells transfected with these plasmids were either permeabilized or not and analyzed by immunofluorescence staining. The transfected cells were all positive after permeabilization with 0.05% Triton X-100, indicating that all tested constructs were expressed (Fig. 2B). As shown above, HA-tagged E1 cannot be detected via immunofluorescence of nonpermeabilized cells. In contrast, VSV-G wild-type (wt) was detected as a rim surrounding the transfected nonpermeabilized cells. Interestingly, the chimera E1_ecto_-VSV-G_TMD_ (pYM-56) was detected on the cell surface. In contrast, the fusion protein VSV-G_ecto_-E1_TMD_ showed no cell surface expression and was completely retained within the cell, similar to HA-tagged E1 wt (Fig. 2B). This result clearly indicated that the proposed TM anchor of the pestiviral E1 functions as an intracellular localization signal; in other words, the TM region is responsible and obviously sufficient for the retention of E1.

The E1_ecto_-VSV-G_TMD_ (pYM-56) chimera was also analyzed via confocal microscopy to investigate its intracellular localization. Expression plasmid pYM-56 was cotransfected with pDsRed-ER or pDsRed-Golgi into BHK-21 cells, respectively. As shown in Fig. 2C, when the transmembrane region of E1 was replaced by that of VSV-G, this chimera was detected only on the cell surface. Neither colocalization with ER nor Golgi apparatus was observed (PCCs of 0.383 and 0.407, respectively), so that the intracellular localization was completely different compared to HA-tagged E1 wt (Fig. 2C) and very reminiscent of VSV-G, a typical plasma membrane protein, known to be almost exclusively found on the cell surface.

To compare the cellular localization of the chimeric proteins with the parental wt proteins (VSV-G and E1), we performed colocalization studies with the wt and chimeric proteins. Cells were cotransfected with the plasmid expressing wt VSV-G and pYM-13 coding for HA-E1 wt. Without permeabilization, only VSV-G wt was detected on the cell surface. After permeabilization with 0.05% Triton X-100, the HA-tagged E1 wt was detected, but there was no colocalization between the two proteins (PCC 0.463) (Fig. 2D, upper two rows of pictures). In contrast, a perfect colocalization, with predominant localization on the cell surface of the E1_ecto_-VSV-G_TMD_ chimera encoded by pYM-56 with wt VSV-G, was observed under both nonpermeabilized (PCC 0.867) and permeabilized conditions (PCC 0.922) (Fig. 2D, middle two rows of pictures). Importantly, the VSV-G_ecto_-E1_TMD_ chimera pYM-57 showed an absence of cell surface expression and was completely retained within the ER, colocalized with HA-tagged E1 (PCC 0.922) (Fig. 2D, lowest two rows of pictures). These results confirmed that the transmembrane domains of the proteins determine their localization with no impact of the ectodomains and with the E1 TM provoking ER localization.

### The ER retention signal is located within the middle part of the E1 transmembrane domain.

**FIG 3:**
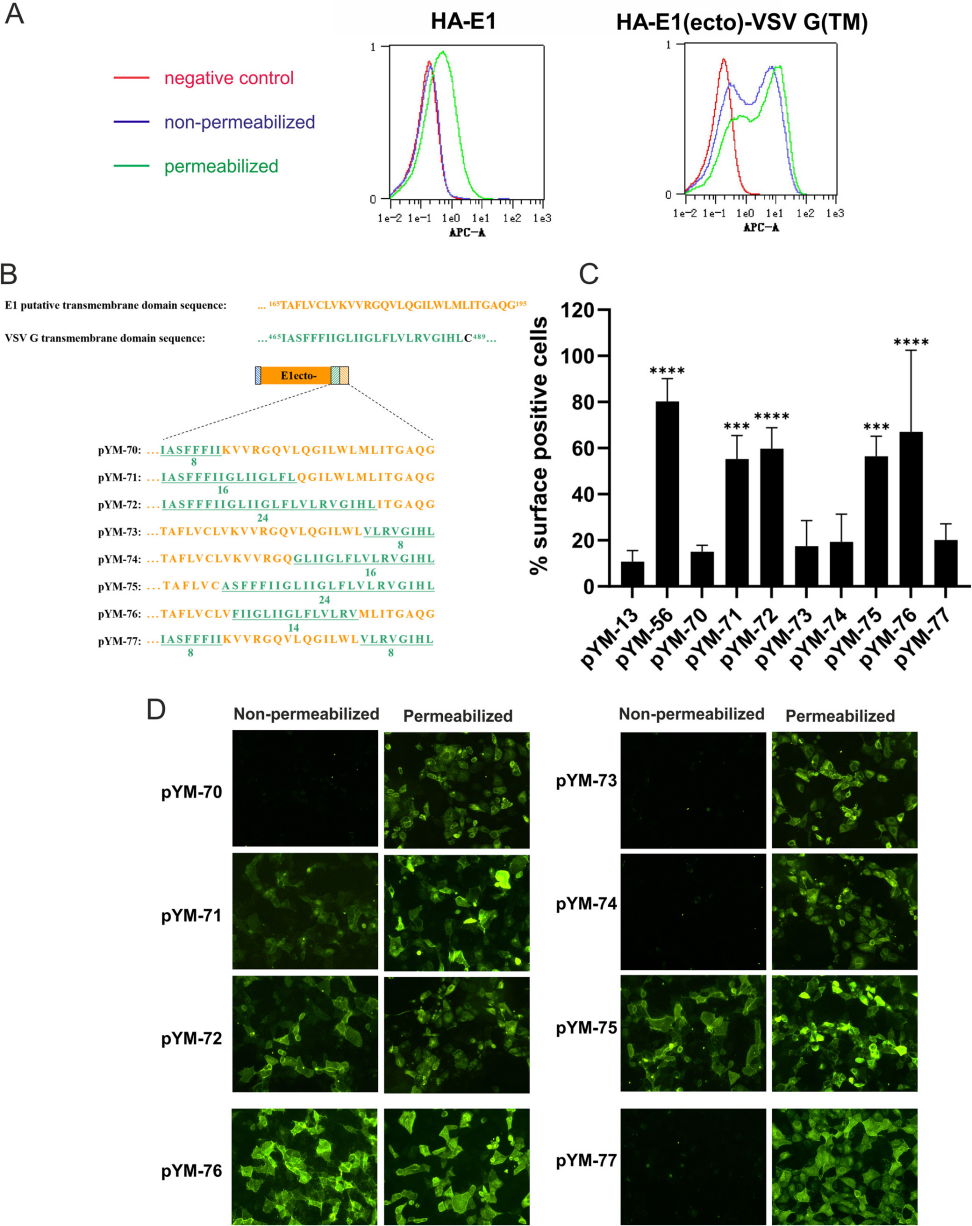
The ER retention signal resides within the middle part of the E1 transmembrane domain. (A) As a control for cell surface expression analysis via flow cytometry, HA-tagged E1 wt (construct pYM-13) or a chimera composed of HA-tagged E1 ectodomain and VSV-G TM region (construct pYM-56) was expressed in RK-13 cells. Transfected cells were fixed by 4% PFA, permeabilized with Triton X-100 or left untreated, immune stained with α-HA/α-mouse FITC, and then analyzed with the MACSQuant. The red peak shows the fluorescence signal of RK-13 cells transfected with pCI empty vector under nonpermeabilization conditions, which served as a negative control; the blue and green peaks show the fluorescence signals of RK-13 cells transfected with the indicated plasmids under nonpermeabilization (blue) and permeabilization (green) conditions. Default settings in MACSQuant were used for flow cytometry analysis, and normalization of peak views was chosen for “height” to make the peaks comparable and the *y* axis remain on an equal scale. (B) Schematic representation of the chimeric transmembrane sequences used in this study. The original sequences of TM domains of both E1 and VSV-G are shown above. The sequences of E1/VSV-G chimeric TM anchors are presented below. (C) Cell surface expression of HA-tagged chimeric proteins analyzed by flow cytometry. The RK-13 cells were transfected with the given expression plasmids. At 24 h posttransfection, the cells were fixed with 4% PFA and stained with α-HA and α-mouse FITC and then analyzed in MACSQuant. The number of surface-positive cells for nonpermeabilized samples is given as the percentage of the number of positive permeabilized cells. Error bars indicate the standard errors of the mean of at least three independent experiments. Data were analyzed using one-way ANOVA test and a Bonferroni *post hoc* test. Asterisks indicate values different from E1 wt. ***, *P* < 0.001; ****, *P* < 0.0001. (D) Presence of cell surface expression of HA-tagged chimeric proteins analyzed by IF. The RK-13 cells were transfected with the corresponding expression plasmids. At 24 h posttransfection, the cells were fixed with 4% PFA, permeabilized or left untreated, and then stained with α-HA/α-mouse FITC and analyzed by fluorescence microscopy.

To confirm the results obtained by immunofluorescence microscopy, we used flow cytometry analyses of transfected cells stained with specific antibodies with or without prior permeabilization. In a first step, the surface expression of the HA-E1 wt (pYM-13) and the E1-VSV-G chimera (pYM-56) was analyzed using flow cytometry. For this purpose, RK-13 cells were transfected in duplicate with the corresponding expression plasmids. The following day, one replicate of each sample was permeabilized with 0.05% Triton X-100 and served as an expression control. The second replicate was processed under nonpermeabilized condition for cell surface expression analysis. Cells transfected with the empty vector pCI served as negative-control cells (red peak shown in Fig. 3A). The shift in signal compared to the negative control in the permeabilized samples showed that all proteins were expressed (green peak). Compared to the pCI control, pYM-56 (E1_ecto_-VSV-G_TMD_), transfected cells showed clear cell surface signals (blue peak). In contrast, HA-tagged E1 wt showed no cell surface expression since there is no significant difference from the pCI negative control. It is worth noting that the exchange of the TM domain to VSV-G TM consistently led to a higher percentage and increased expression of the protein, which led us to normalize our results to the percentage of positive cells in all follow-up flow cytometry experiments. The flow cytometry results match the results of the immunofluorescence analysis (shown in Fig. 2B and 3D).

To further narrow down the area responsible for the ER retention of E1, the ectodomain of E1 (residues 1 to 165) was fused to a series of artificial chimeric transmembrane sequences (Fig. 3B). Since the complete replacement of the E1 TMD by the VSV-G TMD resulted in cell surface expression of E1, the strategy behind this study was a progressive partial substitution of the putative E1 TMD region by the TMD sequence from VSV-G. Replacements started from either the N-terminal or the C-terminal ends. It is worth noting that we kept the total length of the original E1 sequence. For instance, in the pYM-70 construct the sequence coding for the first 8 residues of the E1 TMD (TAFLVCLV) was replaced by the sequence coding for the first 8 aa (IASFFFII) of the VSV-G membrane anchor. The rest of the constructs were made in the same manner. The TM sequences of the chimeric constructs are shown in Fig. 3B. It is worth noting that the last residue in the transmembrane region of VSV-G, the cysteine residue at position 489, was excluded in this study to prevent unpredictable effects from disulfide bond formation. Cell surface expression of these chimeras was investigated by immunofluorescence (IF) and flow cytometry.

The corresponding samples were processed as described in Fig. 3A. Flow cytometry was used to determine the percentage of the cells with E1 on the surface. As stated earlier, we observed a higher transfection and expression rate in the E1_ecto_-VSV-G_TMD_ (pYM-56)-transfected cells. Two-way analysis of variance (ANOVA), followed by Šídák multiple-comparison test of the data for the permeabilized cells, showed that pYM-56 (E1_ecto_-VSV-G_TMD_) and, to a lesser extent, pYM-72 were significantly better expressed than HA-E1 (pYM-13). We therefore used the normalization to compensate for any effects due to this. The percentage of positive cells in nonpermeabilized samples was normalized to their respective permeabilized transfection control and calculated over several experiments. Means and standard deviations (SD) are shown (Fig. 3C). A one-way ANOVA with a Bonferroni *post hoc* test was used to analyze differences compared to HA-tagged E1 wt (pYM-13). pYM-56 (E1_ecto_-VSV-G_TMD_) served as a positive control for the protein expressed on the surface. pYM-13 (HA-E1 wt) was used as a standard for the wt situation (Fig. 3C**)**. pYM-70, pYM-73, and pYM-74 showed no statistically significant differences from pYM-13 (HA-E1 wt). These results match the results obtained by immunofluorescence analysis (Fig. 3D), showing that those three fusion proteins were completely retained in the cell. In contrast to this, pYM-71, pYM-72, and pYM-75 showed a very clear cell surface expression in nonpermeabilized cells and thus differed significantly from the pYM-13 (HA-E1 wt) control, which again was also seen in the IF analysis (Fig. 3D). These results demonstrate that 8 aa from the N-terminal end and 16 aa from the C-terminal end of the putative E1 TMD can be replaced without loss of the ER retention. Interestingly, the fluorescence signal of pYM-71-transfected cells under nonpermeabilized condition seemed to be not as strong as that of pYM-72, indicating that smaller amounts of the fusion protein expressed from pYM-71 might be present on the cell surface. However, this indication was not supported by the flow cytometry results (compare Fig. 3C and D) and might be in part due to the fact that pYM-72, as well as E1_ecto_-VSV-G_TMD_ (pYM-56), showed better transfection and expression.

The IF and flow cytometry analyses of the E1/VSV-G fusion proteins indicated that the ends of the E1 transmembrane domain are not important for its retention. Fusion proteins started to show significant presence at the cell surface only when the original middle part (K174-L187) of the E1 TM region was replaced by the VSV-G sequence. This indicated that the retention signal or at least critical residues for the retention are present within this middle part.

To characterize the effect of the middle area (from K174 to L187) of the E1 TMD domain on the ER retention of E1, two further chimeras were generated (Fig. 3B). In pYM-76, the K174 to L187 of the E1 TM domain was completely replaced by VSV-G sequence (F470-V484), while the rest still displayed the original E1 sequence. The other construct (pYM-77) still contained the original E1 TM domain middle sequence, flanked on both sides by 8 aa of the VSV-G TM sequence.

As shown in Fig. 3C and D, both flow cytometry and IF data demonstrated that cell surface expression is detectable only for the fusion protein encoded by pYM-76, in which the middle part of the E1 TMD sequence was replaced by the VSV sequence. Thus, the middle 14 aa of the E1 TM region are necessary and sufficient for the retention of E1.

### The polar amino acids in the middle part of the TM domain play an essential role in ER localization of E1.

**FIG 4:**
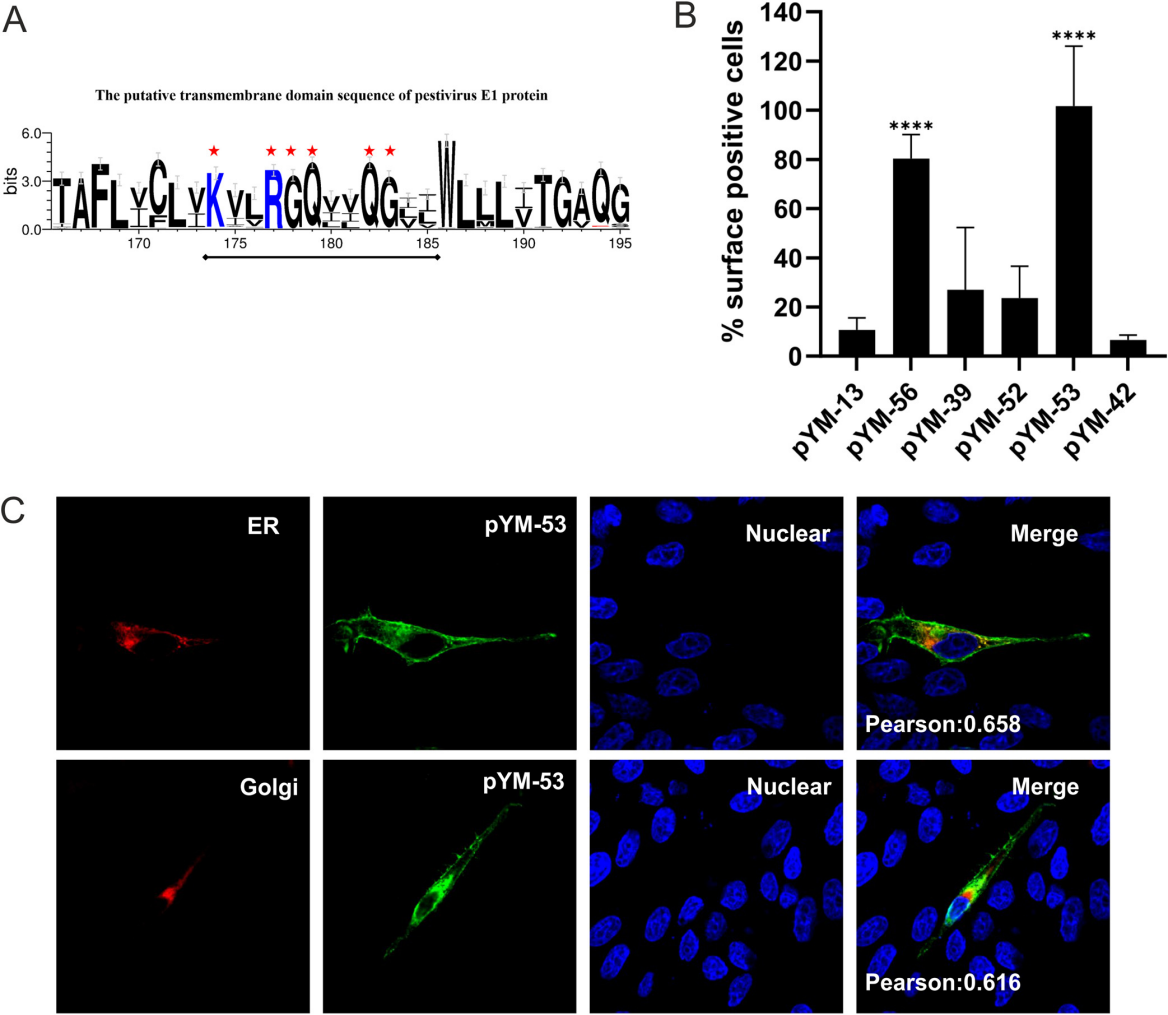
Six polar residues of E1 TM domain are important for ER retention of E1. (A) Conservation of amino acid sequences in the putative TM region of pestiviral E1. The sequence logo was generated by WebLogo 3 web application (http://weblogo.threeplusone.com/create.cgi) and demonstrates the alignment of 68 pestivirus E1 sequences throughout pestiviral species A to K in one-letter code. The size of the letters in the sequence logo corresponds to the degree of conservation. Six highly conserved polar residues in the middle of the sequence are highlighted with red asterisks. For not fully conserved residues, the height of symbols within the stack represents the relative frequency of each amino acid at the corresponding position. (B) Cell surface expression of HA-tagged E1 mutants analyzed by flow cytometry. The RK-13 cells were transfected with the given expression plasmids and analyzed as described in legend to Fig. 3. The plasmids used include pYM-13 (HA-E1), pYM-56 (HA-E1_ecto_-VSV-G_TMD_), pYM-39 (HA-E1 with K174A and R177A), pYM-52 (HA-E1 with K174A, R177A, G182A, and Q183A), pYM-53 (HA-E1 with K174A, R177A, Q178A, G179A, G182A, and Q183A), and pYM-42 (E1 Q182A and G183A). (C) Subcellular localization of the pYM-53-derived HA-E1 mutant containing six mutations (K174A, R177A, G178A, Q179A, Q182A, and G183A) in the TM domain analyzed via coexpression with pDsRed-ER or pDsRed-Golgi, respectively. At 24 h posttransfection, the cells were fixed by 4% PFA, permeabilized with 0.05% Triton X-100, and stained with specific antibodies against HA (green). Compartments (ER or Golgi) are indicated in red. Nuclei were stained with DAPI (blue).

The conservation of the amino acid sequence of the E1 TM domain throughout all species of pestiviruses was analyzed by WebLogo 3 web application (Fig. 4A). Several polar residues are fully conserved, including four noncharged residues (glycine and glutamine) and two positively charged residues (arginine and lysine). Those fully conserved residues were hypothesized to be functional for the retention in analogy to other envelope proteins from pestiviruses and related viruses which are retained because of polar residues in the TM region (15, 17, 20–23).

To hunt for the critical site essential for E1 retention, a mutagenesis analysis was carried out, in which selected polar amino acids were substituted or deleted. The corresponding expression plasmids (Table 1) were transfected into RK-13 cells and the surface presence of the proteins was investigated by both IF and flow cytometry. While all single mutations led to slight changes in the surface presence of the HA-tagged E1 mutants, none of the tested substitutions affected the retention of E1 strongly (data not shown). Therefore, E1 variants with double mutations were generated (Table 1). Surprisingly, the IF and flow cytometry data still showed that those selected mutations did not increase the surface presence of the mutated proteins (Fig. 4B and data not shown). It was reported that the length of TMDs of membrane proteins can affect the intracellular trafficking and sorting of proteins, which is supposed to be connected with the different thickness of membranes in the cellular compartments (24–26). Based on this theory, we made two hydrophobic sequence insertions at T166/A167 or G178/Q179 site to extend the length of the TM region of E1. Surprisingly, no significant subcellular localization change was observed (data not shown). In conclusion, single or double substitutions, or even fragment insertion in the TM region of E1, could not lead to a significant increase in the surface expression, indicating that the retention signal of E1 is apparently not dependent on single polar amino acids or the length of the TM domain.

**TABLE 1:** Mutagenesis and insertion analysis for conserved residues in TMD of E1^*a*^

Single mutation(s)	Double mutations	Insertion
K174: A/E/Δ	K174A and R177A (pYM-39)	(i) Between T166 and A167 LLALLA insertion
R177: A/E/K/Δ	R177E and Q182A	(ii) Between G178 and Q179 LLALLA insertion
G178: L	G178L and G183L	
Q179: N/E/A/Δ	Q182A and G183A (pYM-42)	
Q182: N/A/Δ		
G183: L		

a The original amino acid is given in the first column; the positions in the E1 protein, followed by the different substitutions that were tested, are also shown in one-letter code. Δ = deletion of amino acid.

As a next step, two new E1 mutants containing four (pYM-52) and six mutations (pYM-53) were generated and tested in RK-13 cells via flow cytometry. As for all flow cytometry experiments described above, the percentage of positive cells in nonpermeabilized samples was normalized to the percentage of positive cells in the permeabilized sample to account for potential differences in transfection or expression efficacy, even though the samples showed no significant differences in permeabilized samples as tested by two-way ANOVA. As shown in Fig. 4B, surface expression of the protein with four mutations (K174A, R177A, G178A, and Q179A) in the TMD did not differ significantly from the results with pYM-13 (HA-E1 wt), showing hardly any surface expression. However, when all six conserved polar residues were replaced by alanine (pYM-53), the mutated E1 variant shows plasma membrane localization.

To investigate the subcellular localization of the pYM-53 derived protein, the pDsRed-ER or pDsRed-Golgi plasmids were coexpressed with the HA-tagged E1 mutant. As shown in Fig. 4C, colocalization analysis of pYM-53 with the ER/Golgi compartment markers demonstrated that the E1 retention defective mutant presented a plasma membrane localization but was also partially located in the ER (PCC 0.658) and the Golgi compartment (PCC 0.616). The partial ER/Golgi localization of pYM-53 suggested that the retention signal of E1 has been destroyed, so that this E1 retention defective mutant distributed on the secretion pathway. Compared to the entire cell surface presence of the E1-VSV-G chimera (pYM-56: E1_ecto_-VSV-G_TMD_), pYM-53 seemed to be different. Since there are some E1 retention defective mutants still detectable in the ER, export of the mutant protein is either not complete or occurs more slowly than for VSV-G. Taken together, these data showed that the fully conserved polar amino acids in the middle part of the E1 TM domain (Lys174, Arg177, Gly178, Gln179, Gln182, and Gly183) play an essential role in intracellular retention and ER localization of E1. Obviously, none of these six residues is *per se* crucial, so only the presence of a variable selection of these residues is needed for retention.

### Effect of selected mutations in E1 on the replication of BVDV strain CP7.

**FIG 5:**
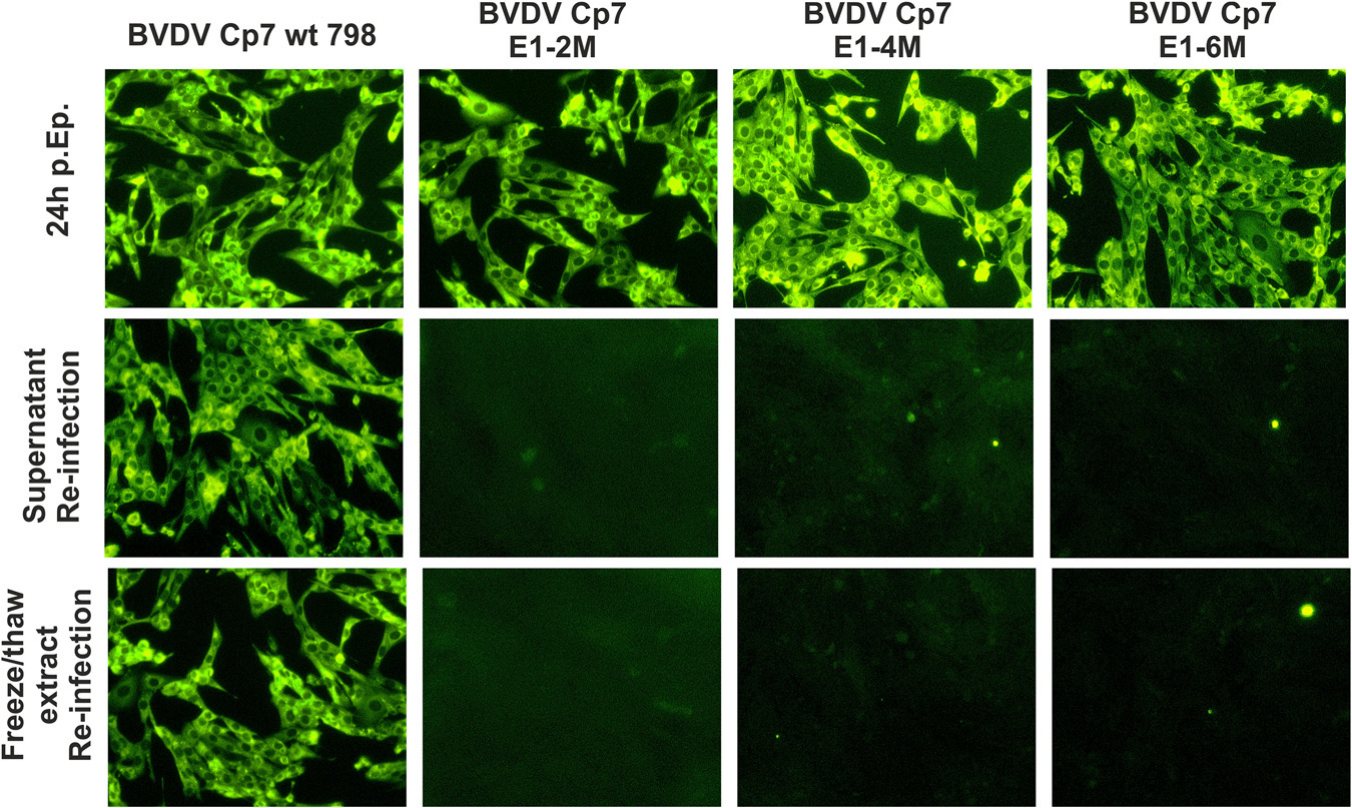
Effect of the selected mutations in E1 on the replication of BVDV strain CP7. Top row (24h p.EP.): MDBK-B2 cells were first transfected by electroporation with the RNAs transcribed from the given plasmids. The corresponding plasmids are mentioned at the top. The plasmid BVDV-CP7 E1-2M contains the same mutations as pYM-39, and the 4M and 6M versions are equivalent to pYM-52 and pYM-53, respectively. One day after EP, the cells were fixed with 4% PFA and permeabilized with 0.05% Triton X-100. The viral protein NS3 was detected with the primary antibody Code4 and α-mouse FITC. Middle row: supernatant reinfection after electroporation. At 2 days after EP, the supernatant from the electroporated cells was added to fresh MDBK cells for reinfection. At 2 days after the reinfection, the SN reinfected cells were checked again for the presence of NS3 by indirect immunofluorescence. Bottom row: freeze/thaw extract reinfection after electroporation. At 2 days after EP, the supernatant of the electroporated cells was removed, the electroporated cells were lysed via three times complete freeze/thaw cycling, and cell extract was added to the fresh MDBK cells for a reinfection test. At 2 days after the reinfection, the cells were checked again for the presence of NS3 by indirect immunofluorescence.

The data presented above could not explain the strong conservation of the hydrophilic residues in the E1 TMD. To get more information, we introduced the above-mentioned mutations K174A/R177A (E1-2M), K174A/R177A/G178A/Q179A (E1-4M), and K174A/R177A/G178A/Q179A/Q182A/G183A (E1-6M) into the BVDV CP7 full-length cDNA construct pA/BVDV (27), respectively, and tried to recover infectious viruses upon *in vitro* transcription and electroporation of viral genome-like RNA. An aliquot of the transfected cells seeded in a 3-cm dish was tested by immunofluorescence with the BVDV-specific antibody Code4 the day after electroporation. Numerous positive cells were detected for all three mutants in contrast to the mock-treated control (Fig. 5, upper row of pictures). This result proves that the introduced RNA was able to replicate autonomously since we do not see proteins translated from input RNA in our system. After longer incubation times, the positive cells were mostly rounded due to the cytopathic phenotype of BVDV CP7 (not shown). RNA replication of a cytopathic BVDV was shown to be sufficient for induction of cell death (28). In contrast to the wt positive control, attempts to infect fresh cells with supernatant or freeze/thaw extract of transfected cells failed even after a longer incubation of transfected cells (Fig. 5, medium and lower row of pictures). We therefore concluded that the introduced mutations prevented generation of infectious viruses. The replication time from transfection until cell death in consequence of the cytopathogenic effect of RNA replication was obviously not sufficient to allow (pseudo)reversion via mutation of viral RNA, as often seen for mutants of noncytopathogenic pestiviruses before (29).

### Membrane topology of E1.

The N terminus of E1 is generated by signal peptidase cleavage at the unusual E^rns^ membrane anchor/E1 site, so that the N terminus of E1 should be located in the ER lumen (11, 13). The hydrophobic region at the C terminus of E1 is too long for a normal single span transmembrane domain, so the membrane topology of the mature E1 protein is difficult to predict, and studies on the membrane topology of E1 are still missing. To investigate the membrane topology of transiently expressed E1, indirect immunofluorescence analysis after selective permeabilization of cells was carried out. The cells were fixed with 4% paraformaldehyde (PFA) and then either incubated with 0.05% Triton X-100 for 30 min or treated with 5 μg/ml digitonin for 15 min at 4°C. The former procedure results in the permeabilization of both the plasma membrane and the compartment membranes, whereas the digitonin treatment leads to only plasma membrane permeabilization, while the compartment membranes remain intact and represent an insurmountable barrier for the antibodies used for the detection of proteins. Triton X-100 permeabilization served as a control for the expression and antibody binding, since the target proteins can be detected in all areas of the cell. In contrast, only proteins on the cell surface and in the cytoplasm can be detected in the digitonin permeabilized preparations.

**FIG 6:**
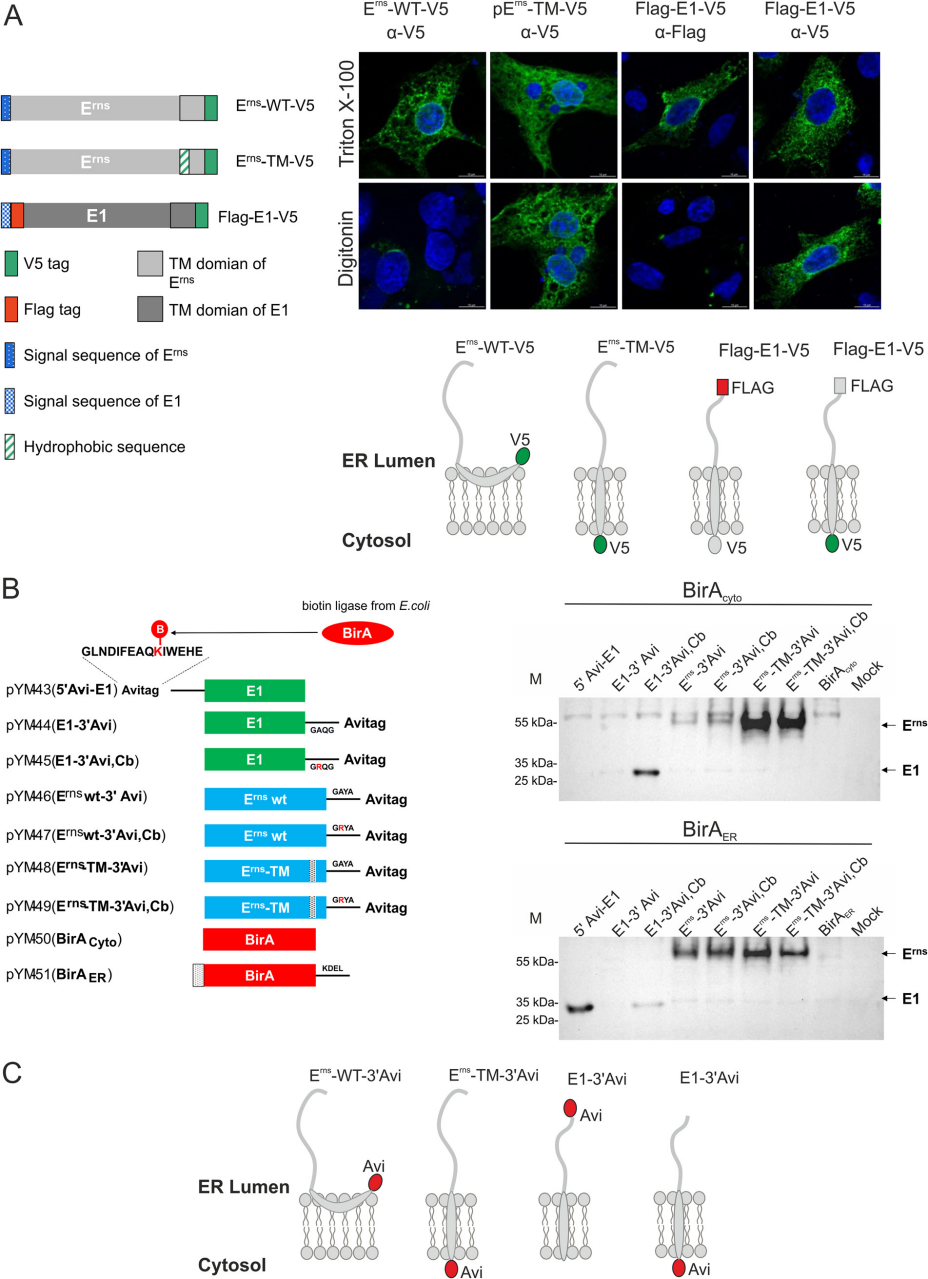
Membrane topology of E1. (A) The upper part shows a schematic representation of constructs used in an immunofluorescence analysis: E^rns^ wild type with C-terminal V5 tag, E^rns^ TM with leucine stretch instead of the amphipathic helix and C-terminal V5 tag (18), and double-tagged E1 variant with an N-terminal Flag tag and a C-terminal V5 tag. On the right side, the results of immunofluorescence analyses are shown. BHK-21 cells were transfected with the indicated plasmids and fixed with 4% PFA on the following day. Cell membranes were permeabilized with Triton X-100 (all membranes) or the plasma membrane was selectively permeabilized with digitonin, followed by staining with specific antibody combinations: E^rns^, α-V5, α-mouse Alexa-Fluor-488; E1, α-FLAG, α-mouse Alexa-Fluor-488 or α-V5, and α-mouse Alexa Fluor 488; and nucleus, DAPI (blue). Below the micrographs are schematic representations of the membrane topology of the analyzed proteins. (B) As a second approach, *in vivo* labeling of proteins via BirA-mediated biotinylation of Avi tag-labeled proteins was conducted. On the left are constructs used in this section. The Avi-tagged proteins were transiently expressed in RK-13 cells cotransfected with pYM-48 (BirA cytosol, upper right) or pYM-49 (BirA ER, bottom right), and the expression products were analyzed for biotinylated proteins via Western analyses with α-streptavidin PO. (C) Schematic representation of membrane topology of Avi-tagged constructs.

In this study, two variants of the BVDV glycoprotein E^rns^ with known topology were used as controls for the correct selective permeabilization (18). The plasmid construct pB11 encodes the BVDV CP7 wt E^rns^ with a C-terminal V5 tag. All the epitopes of this protein are known to be on the luminal side of the ER and should therefore not be detectable after digitonin permeabilization. pB154 is the other expression plasmid that expresses a variant of E^rns^ with a hydrophobic leucine stretch replacing the original amphipathic helix at the C terminus of E^rns^, also followed by a carboxy-terminal V5 tag. The exchange of the amphipathic helix for a hydrophobic region leads to a transmembrane domain resulting in exposure of the V5 tag on the cytosolic side of the ER membrane, and it can therefore also be detected after digitonin permeabilization (Fig. 6A) (18).

Both E^rns^ variants were expressed in BHK-21 cells. Upon selective permeabilization of the transfected cells, the E^rns^ proteins showed the expected pattern, which confirmed that the assay was done correctly (Fig. 6A). To determine the membrane topology of E1 expressed in the absence of any other viral protein, a double-tagged E1 variant containing an N-terminal Flag tag and a C-terminal V5 tag was used. As shown in Fig. 6A, both tags could be detected in the Triton X-100-permeabilized cells with specific antibodies, which indicated that the proteins were successfully expressed and accessible for antibody binding. After permeabilization with digitonin, the V5 tag at the C terminus of E1 could be detected, whereas the Flag-specific antibody did not give a specific signal. These results showed that the N terminus was as expected on the luminal side of the ER and the C terminus was in the cytosol. Thus, the selective permeabilization assay supported the conclusion that pestiviral E1 is a type I transmembrane protein that has a N-terminal ectodomain in the ER lumen and a C-terminal transmembrane anchor.

To further confirm this conclusion, a sensitive and selective biotechnology approach called the Avi-tag biotinylation assay was used. The Avi tag is a short peptide of 15 aa (GLNDIFEAQKIEWHE) which can be covalently attached to biotin in the presence of *Escherichia coli* biotin ligase (BirA). The biotin moiety bound to the Avi tag can then be detected via (strept)avidin. It is known that the interaction between biotin and streptavidin or avidin is very strong, sensitive, and selective, so (strept)avidin-biotin binding has been widely used in molecular biology research (30–33).

A short sequence coding for the Avi tag peptide as a target for site-specific biotinylation was fused to the region coding for the N terminus or C terminus of target proteins. By coexpression with the modified BirA biotin ligase, this Avi tag can be labeled depending on the localization of the Avi tag at the target protein and on the subcellular distribution of BirA. The advantage of this system over the IF/differential permeabilization approach is that the labeling occurs under native conditions before the cell is destroyed.

The Avi tag was fused to either the N terminus or C terminus of E1. To ensure that the Avi tag at the C terminus cannot be cleaved off, we introduced a mutation Ala to Arg at the −3 position of the SP cleavage site at the C terminus of E1, thereby blocking the von Heijne motif of the SP cleavage site at the E1 carboxy terminus (constructs “Cb” for cleavage blocked in Fig. 6B) (10). For control, constructs coding for E^rns^ wt and E^rns^ TM with the carboxy-terminal Avi tag were also established. Also, in these constructs, the cleavage site at the C terminus of E^rns^ was blocked. In this study, we constructed two types of BirA expression plasmids. The plasmid construct pYM-48 (BirA_Cyto_) encoded biotin ligase (BirA) without signal sequence so that all the expressed biotin ligase should be located in the cytosol. In contrast, the plasmid pYM-49 codes for a BirA version with an amino-terminal signal sequence (signal sequence of BVDV E^rns^: MALLAWAVITILLYQPVAA) and the well-characterized ER retention signal KDEL at the C terminus. Theoretically, this modified BirA should predominantly be located in the ER.

RK-13 cells were cotransfected with constructs BirA_Cyto_ or BirA_ER_ and the plasmids coding for the Avi-tagged E1 or E^rns^ proteins, respectively. On the following day, cell lysates were prepared and separated by SDS-PAGE. Western blot analysis was performed using peroxidase-coupled avidin (avidin-PO) for detection of biotinylated proteins. As shown in Fig. 6B, the control E^rns^-TM-Avi was detected as the dominant band when coexpressed with BirA_Cyto_, regardless of whether the Cb version was used or not (lanes 6 and 7). Only a very faint band was visible for E^rns^ wt-Avi, the version with the C-terminal amphipathic helix. This indicated that BirA_Cyto_ was at least predominantly expressed in the cytoplasm, since only the tag accessible from the cytosol was biotinylated. For the E1-Avi tag fusion protein samples, only the Avi tag located at the C terminus of E1 with the cleavage site block could be biotinylated, demonstrating that the C terminus of E1 is located in the cytosol.

When the Avi-tagged constructs were coexpressed with BirA_ER_, both E^rns^ wt-Avi and E^rns^ TM-Avi were biotinylated (Fig. 6B, lanes 4, 5, 6, and 7). In light of the published and above-presented data, this result suggested that either the C terminus of E^rns^ TM (3′-E^rns^-Avi) is in part of the cases exposed in the ER lumen, a point that would not have been detected in previous analyses, or the BirA expression construct after introduction of a signal sequence and ER retention signal is not exclusively present in the ER. The latter could be the reason why there is still a very slight band for E1 with C-terminal tag (3′-E1-Avi) detected after coexpression with BirA_ER_. The N-terminal Avi-E1 (5′-Avi-E1) can be biotinylated in the presence of BirA_ER,_ yielding a much stronger signal than the carboxy-terminally tagged version, which clearly shows that the N terminus of E1 is located in the ER lumen (Fig. 6B, right part). These results, together with the data previously collected via the selective permeabilization assay, strongly support the conclusion that E1 in its mature form represents a typical type 1 membrane protein (as schematically illustrated in Fig. 6C). Due to the length of the hydrophobic sequence at the C terminus of E1, the residues located within the lipid bilayer and thus representing the TM-domain cannot be predicted but would have to be identified experimentally. The same is true for the E2 membrane anchor.

### The TM domain of pestiviral E1 forms a hairpin structure before signal sequence cleavage.

**FIG 7:**
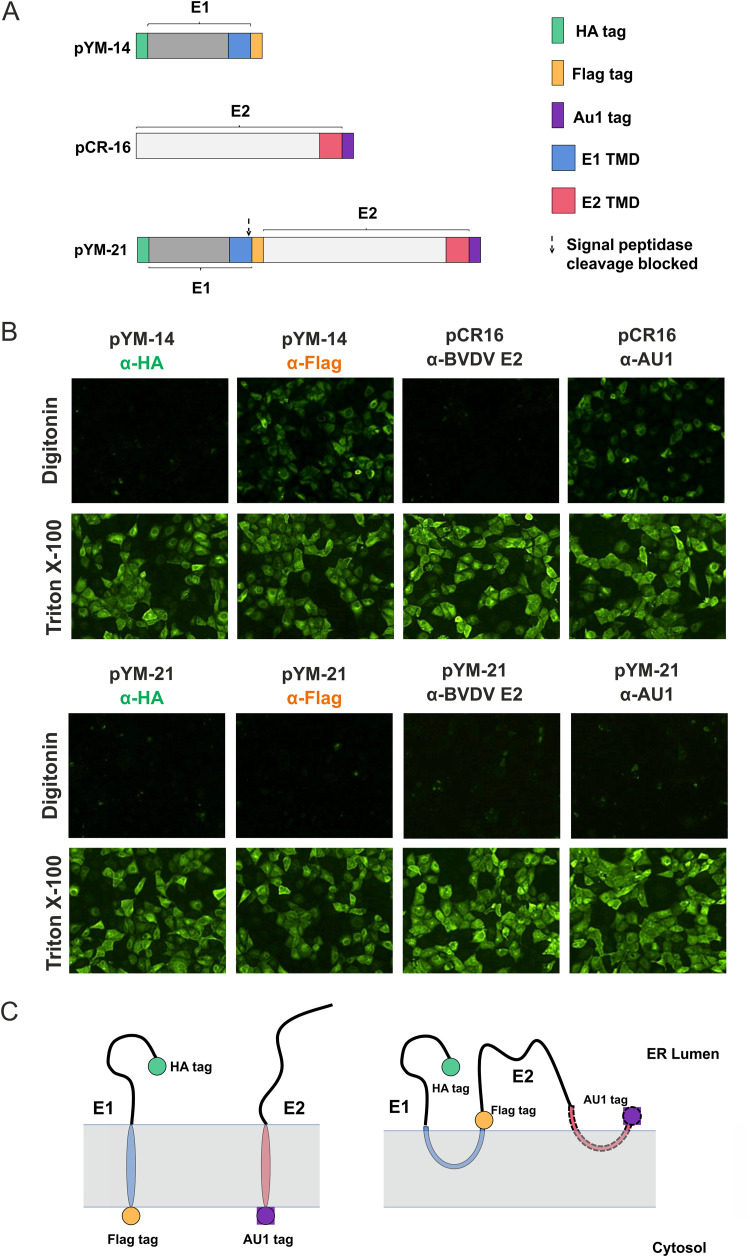
The TM region of pestiviral E1 forms a hairpin structure before signal sequence cleavage. (A) Schematic representation of constructs used in this section. (B and C) RK-13 cells were transfected with the given expression plasmids and fixed with 4% PFA on the following day. Either all the cell membranes were permeabilized with Triton X-100, or only the plasma membrane was permeabilized with digitonin. Protein detection was done as indicated. E1: α-HA, α-FLAG, α-mouse Alexa-Fluor-488; E2: α-E2 (BVDV MIX anti E2 ectodomain), α-AU1, α-mouse-FITC. (C) A schematic representation of the membrane topology of the different proteins is shown below the pictures, with the membrane represented by light gray transparent bars.

Normally, the C-terminal part of viral transmembrane proteins in polyproteins can be divided into two parts: the first part is a TM segment that generally consists of 16 to 25 mostly nonpolar residues, and the second one is a short hydrophobic sequence (7 to 12 aa) serving as the signal sequence, which directs the translocation of the following precursor. The presence of a signal sequence in the second half of the C terminus of the TM domain of E1 does not fit with a single membrane-spanning topology. Since pestiviral envelope proteins are synthesized as a polyprotein, it is conceivable that the membrane topology of the polyprotein precursor should be different from that found after the signal sequence cleavage occurred since the C terminus of E1 in the polyprotein should be located on the ER-luminal side to allow translocation of the downstream E2. We therefore wanted to analyze the membrane topology of the C terminus of E1 in the precleavage state. To mimic this state, we conducted analyses under conditions where the signal sequence cleavage was hampered. To achieve this, construct pCR-17 coding for the BVDV CP7 E1-E2 proteins was used. We introduced a mutation (Ala to Arg) at the C terminus of E1 at position −3 of the cleavage site to block the signal sequence cleavage. For the detection of the N and C termini of E1, an HA epitope (YPYDVPDYA) and a Flag tag sequence (DYKDDDDK) were fused to its N or C terminus, respectively. In addition, E2 was also tagged at the C terminus with an AU1 epitope (DTYRYI) for tracing whether the C terminus of E2 is still accessible from the cytosol when cleavage at the E1/E2 site was blocked (Fig. 7A). This newly made plasmid construct was named pYM-21. To analyze the membrane topology of this uncleaved E1-E2 precursor, we again used the selective permeabilization assay.

As a control, cells permeabilized with 0.05% Triton X-100 were analyzed in parallel. In this experiment, pYM-14 (ss-HA-E1-Flag) and pCR-16 (ss-E2-AU1) were cotransfected to mimic the situation after the signal sequence cleavage. A mixture of monoclonal antibodies (MAbs) directed against BVDV CP7 E2 called BVDV-Mix was used for the detection of the E2 ectodomain. As shown in Fig. 7B, the C terminus of both E1 and E2 were accessible to the respective antibodies in the digitonin permeabilized cells when E1 and E2 were expressed as individual proteins from the cotransfected plasmids. This result fits well with the results and the conclusions described above. Surprisingly, when the fusion construct with the blocked cleavage site was analyzed, all of the epitopes were only accessible to their respective antibodies in Triton X-100 but not in digitonin-permeabilized cells (Fig. 7B). These data indicate that the C terminus of E1 is located on the luminal side of the ER membrane, most likely adopting a double membrane-spanning structure in the absence of signal sequence cleavage. Moreover, this also suggests that before the cleavage between E1 and E2 has occurred, the C terminus of E2 cannot adopt a transmembrane configuration in the ER membrane, the final topology of the mature E2 protein. The membrane topology of the sequence preceding the E2 C terminus should most likely be equivalent to that shown here for the precleavage state of E1, but since we have not analyzed this point in detail, this highly hydrophobic sequence is shown here as a red line with a dotted contour in the membrane (Fig. 7C).

## DISCUSSION

Viruses have to take advantage of the protein biosynthesis machinery of the host cells for their own protein synthesis and processing via the conventional protein modification and trafficking pathway. Accordingly, viral envelope proteins need to have a signal peptide to be translocated into the ER, where the co- or posttranslational modification starts, followed by delivery to their final destination via the secretory route. Some viral envelope proteins remain within defined intracellular compartment(s) because they contain a respective localization signal. This leads to accumulation of the proteins at the site of intracellular budding of progeny viruses. Assembly and budding can occur at the ER (e.g., rotaviruses and hepatitis C virus), the ER-Golgi intermediate compartment (ERGIC) (e.g., coronaviruses and poxviruses), or the Golgi apparatus (e.g., bunyavirus) (22, 23, 34). Other viruses, such as retroviruses or lyssaviruses, bud through the plasma membrane, which also means that their envelope proteins do not contain retention signals (35, 36). The pestiviral envelope proteins E^rns^ and E2 were shown to localize in the ER (16, 17, 20, 21). In addition, an ultrastructural study of pestivirus Giraffe-1 using electron microscopy also showed that ER is the initial cellular organelle for pestivirus assembly and budding (14). These data strongly indicate that pestiviruses bud at the ER, so that their envelope membrane is derived from the host ER membrane.

It has been confirmed that both E^rns^ and E2 contain retention signals of their own (16, 17). For E1, detailed data on retention and intracellular localization have so far not been published. E1 forms covalently linked heterodimers with E2. E1 could therefore be retained in the ER through E2 and its retention signal. However, the HCV envelope proteins E1 and E2 both contain retention signals and, moreover, wild-type pestiviral E1 was able to rescue a retention defect in pestiviral E2, suggesting a separate retention signal in E1 (17, 22, 23). In our study, we showed that there is indeed an ER retention signal present in the transmembrane region of the pestiviral E1 glycoprotein. Thus, the E1-E2 envelope protein complexes of pestiviruses contain two signals for ER retention, which might be necessary to provide enough time for E1 and E2 to form a complex. Both signals should ensure accumulation at the same location to allow heterodimer formation. It could, however, also be that E1 has an additional separate function not connected with the heterodimer and needs a separate retention signal in this context. This would fit with the published data that not all synthesized E2 molecules are found in heterodimers. A smaller amount forms E2 homodimers which are also present in pestivirus virions (5), so that also a certain amount of free E1 has to exist.

The TM regions of the envelope proteins of the members of *Flaviviridae* are multifunctional since they ensure the membrane anchoring and intracellular retention of the proteins, serve as signal sequences for the protein downstream, and are involved in intermolecular interactions such as dimer formation. The TM sequences are usually composed of two hydrophobic sections separated by a short segment containing at least one fully conserved positively charged residue (15, 22, 23). However, there are some differences in the functionality, length, and topology of the TM regions between members of the *Flaviviridae* belonging to different genera. In members of the genus *Flavivirus*, the membrane (M) and envelope (E) protein have TMD that form two antiparallel α-helices (37, 38) and have ER retention signals that can depend on length and nonhydrophobic residues for their ER retention and thus are different from those of members of the genera *Pestivirus* and *Hepacivirus* (39–41). For HCV, publications showed that these conserved positively charged residues play an essential role in the retention and heterodimerization of glycoproteins, as well as in the assembly of the viral particle (15). Similarly, the pestiviral E2 protein contains in its TM domain a conserved arginine that is of major importance for retention (20). However, the cytoplasmic domain of the protein and especially a glutamine in this sequence also had an influence on retention (17). Our experiments revealed that retention of pestiviral E1 is also mediated by the proposed TM domain. Based on the data published on the retention of HCV E1 and E2, as well as pestiviral E2, we started our search for the responsible E1 retention signal looking for positively charged amino acids in the TM domain. Indeed, we found two fully conserved positively charged residues (K174 and R177) located in the middle segment of the TM. However, replacement of these residues, either in single or in double mutants, revealed that these exchanges have nearly no effect on E1 retention (Fig. 4 and data not shown). In addition to the two positively charged residues mentioned above, the respective region of the E1 membrane anchor contains four conserved noncharged polar amino acids that might be engaged in E1 retention. Surprisingly, only mutation of all the six fully conserved polar residues to alanines resulted in the presence of E1 on the cell surface (Fig. 4). This finding indicated that pestiviral E1 may adopt a retention mechanism differing from other envelope proteins of the genera *Hepacivirus* and *Pestivirus*. Since these polar residues are all present in the connecting region, they seem to represent a redundant retention signal that can obviously only be destroyed via concomitant mutation of all polar residues as shown by the 6×Ala mutant. One effect of this unusual configuration is the stability of the intracellular retention of E1, which is resistant against a large variety of changes. Even the introduction of a diacidic “DXE” export signal from the VSV-G cytoplasmic tail to the C terminus of the E1 TM domain did not result in the presence of E1 on the cell surface. This finding suggested that the ER retention signal present in the E1 TM domain is dominant over the diacidic export signal and thus proved the stability and strength of the retention signal of E1 (data not shown).

The glycoproteins E1 and E2 of pestiviruses were identified to be type I transmembrane proteins retained in the ER. Cellular type I transmembrane proteins located in the ER often contain a dilysine motif (e.g., -KKXX, and -KXKXX) in the cytosolic C terminus (42), while luminal ER proteins often have a KDEL sequence (43). The ER retention signals, in principle, can be divided into two types. One can make proteins resident in the ER at steady condition without cycling between the ER and the Golgi apparatus. HCV E1 and E2 contain this kind of retention signal (15, 22, 23, 44). The other is a so-called retrieval signal, which can return the target proteins from the Golgi complex to the ER via COP-I vesicles. The retention signals in both E1 and E2 of pestiviruses most likely belong to the former. KDEL as a retrieval signal returns the cargo from the Golgi apparatus to the ER via a well-characterized specific receptor (45–48). Like many viral (41) envelope proteins, the pestiviral glycoproteins do not contain one of these known retention signals. Thus, the retention mechanism of pestiviral envelope proteins is still not clear at the molecular level.

As mentioned before, members of the genus *Flavivirus* have different cellular retention mechanisms. Yellow fever virus (YFV) also contains retention signals in both premembrane (prM) and envelope (E) protein. As shown for YFV, the mechanism of ER retention of these proteins mainly relies on the length of the transmembrane stretches (41), and mutation of the charged amino acids has no impact on retention and subcellular distribution (39). The retention signal for YFV, as well as dengue virus, E lies in the first helix of the TMD (39, 40). However, at least for YFV, the retention signal in prM is stronger and can compensate for loss of the E retention (41). According to the so-called “lipid-based” rule (49), membrane thickness plays a major role in the ER retention mediated by these TMDs since the lengths of the transmembrane regions of membrane proteins are strongly associated with their locations in different organelles along the secretory pathways (25, 50–52). For HCV, the retention mechanism of E1 and E2 primarily depends on the polar charged residues in the middle parts of their TM domains. The same is true for pestivirus E2. In our study, we showed that six fully conserved polar residues affect the ER retention of pestiviral E1, indicating that this mechanism could be a different variant of the type found in HCV and pestivirus E2. Our findings indicate that retention is achieved by polar residues. TM domains could be quite flexible with regard to the positioning of the polar amino acid in the TM context. If this was also true for pestiviral E2 and HCV E1 and E2, retention might rely more on physicochemical properties of the membrane-interacting TM sequence than on a specific structure or sequence motif interacting with a defined partner molecule. The exact molecular mechanism for the retention behavior of the envelope proteins from pesti- and hepaciviruses still awaits further investigation.

The conservation of the six polar residues in the middle of the E1 TM domain cannot be explained by their function for retention since a significant number of these amino acids can be replaced by alanine without significant effect on intracellular localization of E1. Due to the instability of the genomic sequence in positive-strand RNA viruses, sequence conservation has to rely on selective pressure. Our experiments with mutated viral genome-like RNAs prove that the polar residues indeed have a crucial importance for recovery of infectious viruses, most likely in the context of virus assembly, budding, or the infection process. The data generated thus far cannot distinguish between a block affecting the generation of virus particles, their release, or the subsequent infection of new cells. Further analyses of these processes, including quantification of viral RNA within cells and supernatant, are needed to further evaluate the functions of the six polar residues.

**FIG 8:**
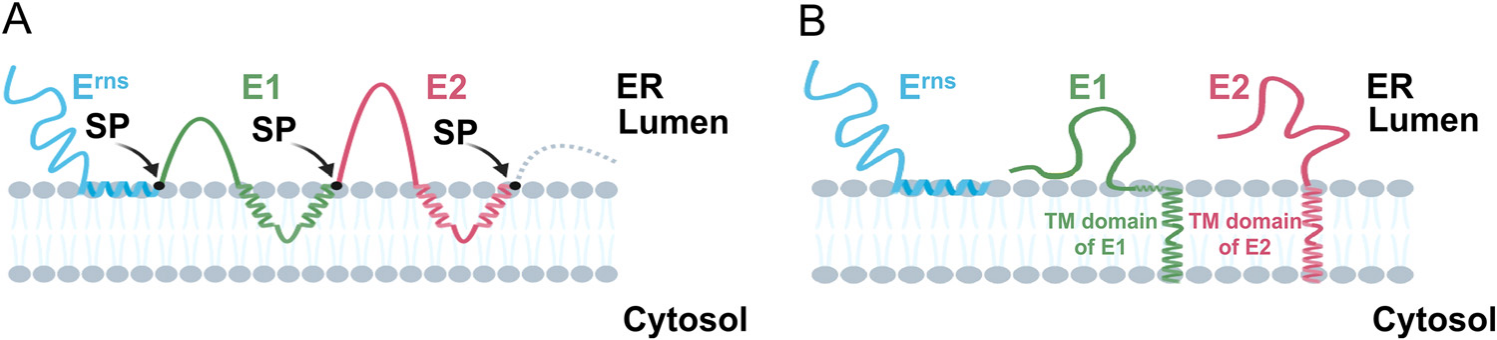
Model of the reorientation of the TM domains of pestiviral envelope proteins following polyprotein processing. (A) The signal peptidase (shown as black arrow) is responsible for the cleavage at the E^rns^/E1 and E1/E2 sites (shown as black dots). Before signal sequence cleavage between E1 and E2, the signal sequence present in the C-terminal half of the TM domain of E1 is oriented toward the ER lumen. Similarly, the TM domain of E2 transiently adopts a hairpin structure to allow the translocation of the following proteins p7 and amino terminus of NS2. (B) After signal peptidase cleavage, the signal sequences present in the C-terminal half of the TM domain of E1 or E2 are reoriented toward the cytosol, establishing a transmembrane configuration spanning the lipid bilayer. The proposed TM domain of E1 is preceded by sequences that were suggested to fold into amphipathic helices able to bind in plane to the membrane surface (19). This hypothetical membrane interaction is also shown in the scheme.

Pestiviral envelope proteins are synthesized as a polyprotein. The signal peptidase is responsible for the cleavage at the E^rns^/E1 and E1/E2 sites, which is possible due to the presence of several membrane-spanning sequences in the structural polyprotein. It is known from other enveloped virus polyproteins that the processing of such membrane-spanning sequences serving as internal signal sequences often combined with a subsequent reorientation of the hydrophobic sequence into a TM domain is essential for establishing the correct membrane topology of mature proteins. Therefore, the membrane topology of E1 before or after the signal peptidase cleavage was analyzed in this study. It has been suggested that one peptide composed of 16 leucines is sufficient to form an α-helix to go through the membrane (51, 52). However, the TM region of integral membrane proteins normally contain stretches of 20 to 25 hydrophobic residues (53, 54). As shown in Fig. 7A, the TM domain of pestiviral E1 contains two hydrophobic stretches of about 10aa, so each hydrophobic region is too short to form a membrane-spanning helix. Accordingly, our analyses revealed a reorientation of the E1 carboxy terminus from an ER lumen location before SP cleavage to a single transmembrane-spanning domain, which in analogy to similar systems is most likely α-helical. Thus, mature pestiviral E1, like E2, adopts a typical type I transmembrane topology after the signal peptidase cleavage, whereas before cleavage a hypothetical banana-like configuration could be adapted, fitting with our results that before cleavage both N and C termini of E1 are located on the ER luminal side (schematic models shown in Fig. 8). A dynamic change in the orientation of the C terminus of E1 was also shown in HCV E1 and E2 (55). A publication on HCV suggested that the extended “hairpin-like” structure of the TM domain is thermodynamically not stable, since those exposed charged residues are not favorable in the hydrophobic membrane environment (23). After the reorientation, the C terminus of the TM domains of the envelope proteins could interact with the viral capsid/RNA complex to initiate the budding of progeny viruses. Moreover, the reorientation generates a stable membrane anchor with the retention signal for the cellular localization of E1 placed in the lipid bilayer.

An important further point concerning the TM domains of E1 and E2 concerns their role in interaction and heterodimer formation of these proteins, as reported for HCV (15, 56). We intend to conduct experiments trying to clarify this point and will present the results in a follow-up publication.

In earlier studies, we observed an interesting phenomenon: if the cleavage at the E1/E2 site in the E^rns^-E1-E2 precursor was hampered, the processing of the E^rns^/E1 site was also inhibited (13). Accordingly, there is a processing hierarchy in E^rns^-E1-E2 so that the E^rns^/E1 site can only be processed after the E1/E2 site. Since the reorientation of the E1 C terminus seems to be dependent on cleavage at the E1/E2 site, it could well be that C-terminal reorientation represents the important step leading to a cleavable E^rns^/E1 site. Since the complete E1 sequence has to be present to allow efficient E^rns^-E1 cleavage one can hypothesize that a conformational change in E1 is the prerequisite for processing. The reorientation of the C-terminal domain could have a significant influence on the structure of E1 and could thus induce such a conformational change. Further experimental work is required to clarify this point.

Thus far, pestiviral E1 has been very poorly analyzed. We provide here a first systematic characterization of its intracellular localization, retention signal, and topology. Our results answer some of the open questions but add new points to a long “to-do list” in order to elucidate the properties and especially functions of this interesting viral protein, such as its interaction and dimerization with E2 as a prerequisite for virus assembly, budding, and infection.

## MATERIALS AND METHODS

### Cells, viruses, and antibodies.

Baby hamster kidney-21 (BHK-21) cells (kindly provided by T. Rümenapf, University of Veterinary Medicine, Vienna, Austria), rabbit kidney-13 (RK-13) cells (CCLV-RIE-0109; FLI), and Madin-Darby bovine kidney-B2 (MDBK-B2) cells (CCLV-RIE-1386, FLI) were grown in Dulbecco modified Eagle medium supplemented with 10 % fetal calf serum (FCS) and nonessential amino acids at 37°C in 5 % CO_2_. For cultivation of MDBK-B2 cells, the FCS was determined to be BVDV-free (pestivirus and antibodies against pestiviruses are not detectable). For immunofluorescence, the cells were seeded on coverslips 1 day before transfection. BVDV strain CP7 was initially provided by E.  J. Dubovi (Cornell University, Ithaca, NY) but was recovered here from pA/BVDV (27).

The primary antibodies used for immunofluorescence and fluorescence-activated cell sorting analyses were BVDV-Mix (MAbs 1a16, 1b8, 1b31, and D5 [57]), f48 (58), Code4 (59, 60), α-HA (anti-HA tag mouse MAb [HA.C5] ab18181; Abcam, Cambridge, UK), α-Flag (Sigma-Aldrich/Merck, Darmstadt, Germany), α-AU1 (Abcam), and α-VSV-G rabbit serum (kindly provided by Stefan Finke, FLI Insel Riems, Germany). The primary antibody used for immunoprecipitation was a polyclonal α-HA serum (anti-HA tag rabbit polyclonal serum ab13878; Abcam). The secondary antibodies used for immunofluorescence were α-mouse FITC, α-rabbit FITC (Dianova, Hamburg, Germany), α-mouse-Alexa Fluor 448, and α-rabbit-Alexa Fluor 448 (all from Life Technology/Thermo Fisher/Invitrogen, Karlsruhe, Germany). Avidin-HRPO was used to detect biotinylated proteins. Secondary antibodies for Western bloting were α-mouse/rabbit-HRPO. Secondary antibodies for flow cytometry were α-mouse FITC, α-rabbit FITC, α-mouse APC, and α-rabbit APC (Dianova).

### Plasmids and cloning.

pB-E^rns^-V5, pB-E^rns^/TM-V5 (18), pcDNA3-VSVg (kindly provided by Stefan Finke, FLI), and pDsRed-ER and pDsRed-Golgi (TaKaRa Bio USA, Inc. [formerly Clontech Laboratories, Inc.]) were used, as well as pCR-13 (BVDV CP7 pCI-E1), pCR-16 (BVDV CP7 pCI-E2-AU1), pCR-17 (BVDV CP7 pCI-E1-E2), and pCR-128 (BVDV CP7 pCI-Flag-E1-V5) (17). pFBD-shortH5-Avi was kindly provided by Timm Harder, FLI, Insel Riems, Germany (33). p798, a full-length infectious clone for BVDV CP7 pA/BVDV (27), was also used.

Plasmid pCI (Promega, Heidelberg, Germany) was used for all expression constructs. DNA fragments were amplified using PCR with specific primers containing restriction sites for cloning into the pCI vector (Promega). Construct pCR13 (pCI-E1) contains the cDNA coding for the entire CP7 E1 sequence, including the corresponding signal sequence (EKALLAWAIIALVFFQVTMG, which serves as a signal sequence for BVDV CP7 E^rns^) (17). pYM-13 is based on pCR-13. An HA tag (YPYDVPDYA) was inserted at the N terminus of E1 downstream of the signal sequence via standard mutagenesis approaches. pYM-43 is based on pYM-13, and a sequence coding for an Avi tag (GLNDIFEAQKIEWHE) replaced the HA tag coding sequence at the 5′ end of the of E1 gene. Similarly, pYM-44 and pYM-45 are based on pCR-13 (pCI-E1) but mutagenized so that they express an Avi tag at the C terminus of E1. To make sure this C-terminal Avi tag cannot be cleaved off by signal peptidase, in pYM-45 an R-to-A mutation was introduced at position −3 of the E1 sequence. For pYM-50, the BirA sequence was amplified from pFBD-shortH5-Avi (33). pYM-51 is based on pYM-50, and sequences coding for the signal sequence of E^rns^ and a KDEL sequence were inserted so that they are expressed at the N or C terminus of BirA, respectively, for its ER retention. pYM-21 is based on pCR-17 (BVDV CP7 pCI-E1-E2), with sequences coding for an HA tag and a Flag tag (DYKDDDDK) introduced at the 5′ and 3′ ends of the E1 gene, respectively. In addition, an AU1 tag (DTYRYI) was inserted at the end of E2.

Point mutations were introduced by PCR-based QuikChange mutagenesis according to the original protocol (Promega). Selected mutated sequences were transferred from E1 expression plasmids to p798 using restriction enzymes supplied by New England Biolabs (Frankfurt, Germany) and Thermo Fisher (Karlsruhe, Germany). Synthetic DNA oligonucleotides were synthesized by Metabion (Munich, Germany).

Cloning and *in vitro* mutagenesis in all plasmids was verified by nucleotide sequencing with a BigDye terminator cycle sequencing kit (PE Applied Biosystems, Weiterstadt, Germany). Sequence analysis and alignments were done using Geneious Prime software (Geneious Prime 2019.2.3) (Biomatters, Ltd., Auckland, New Zealand). Further details on the cloning procedures used here, including primer and plasmid sequences, are available on request.

### Transient protein expression.

At 24 h before transfection, BHK-21 or RK-13 cells were seeded in 24- or 6-well plates. Lipofectamine 2000 (Invitrogen) was used as a normal reagent for the transfection of BHK21/RK13 cells. For this purpose, cells were seeded to be 70 to 90% confluent at the time of transfection. Then, 1 μl of Lipofectamine reagent was diluted in 25 μl of Opti-MEM medium (Invitrogen), and DNA was diluted in Opti-MEM medium to a concentration of 1 μg/μl (for 24-well plates). After the addition of diluted DNA to diluted Lipofectamine 2000 reagent (1:1 ratio), the mixture was incubated at room temperature for 5 min. The DNA-lipid complex was then added dropwise to the cells while slightly shaking the plate. Thereafter, the cells were incubated for 24 h at 37°C, followed by a readout analysis, depending on the aim of the experiments.

### Immunofluorescence and colocalization analysis.

Selective permeabilization was essentially performed as described previously (18). At 24 h after transfection or infection, the cells were fixed with 4 % PFA in PBS for 30 min and either left untreated (nonpermeabilized) or permeabilized in 0.05 % Triton X-100 (all membranes) in PBS or in 5 μg/ml digitonin (only plasma membrane) in 20 mM HEPES (pH 6.9), 0.3 M sucrose, 0.1 M KCl, 2.5 mM MgCl_2_, and 1 mM EDTA for 15 min. Both primary and secondary antibodies were diluted in PBS–10 % fetal calf serum (FCS; E1, α-HA tag), and isotype-specific secondary antibodies were used in double-labeling strategies with different mouse MAbs. Coverslips were then mounted in Mowiol medium with DAPI (4′,6′-diamidino-2-phenylindole), and cells were visualized either on an Axiovert 200M with ApoTome (×63 objective; numerical aperture 1.4) (Zeiss) or on a Leica SP5 confocal laser scan microscope (×63 objective; numerical aperture, 1.4; pinhole, 1 airy unit) with sequential acquisition on the fluorophores in multilabeled samples.

### Flow cytometry.

Cells were transfected in 24 wells in duplicate. After 24 h, the cells were washed with PBS–10 % FCS, and one well per sample was permeabilized with 0.5 % saponin and 1 % FCS in PBS for 5 min at 4°C. Antibodies were diluted in PBS–10 % FCS (E1, α-HA tag), and cells were detached with trypsin and fixed with 4 % paraformaldehyde in PBS. Nonpermeabilized cells were treated the same but without the addition of saponin. Samples were analyzed with a MACSQuant analyzer (Miltenyi Biotec, Teterow, Germany). The surface presence was calculated as follows: (% FITC or APC-positive nonpermeabilized cells)/(% FITC or APC-positive permeabilized cells) × 100. All experiments were repeated several times. The calculated percentages for each experiment were analyzed for significant differences compared to the wt E1 values (pYM-13) using one-way ANOVA and a Bonferroni *post hoc* test.

### Western blotting.

Transfected cells were lysed with reducing 1× SDS sample buffer (120 mM Tris-HCl [pH 6.8], 20% glycerol, 4% SDS, 0.02% bromphenol blue) containing 5 % β-mercaptoethanol, and proteins were resolved in duplicate by SDS-PAGE, transferred to a nitrocellulose membrane (GE Healthcare/Fisher Scientific, Schwerte, Germany), and detected using either avidin-HRPO (Invitrogen) or specific primary (E1, α-HA tag) and secondary (α-mouse/rabbit-PO) antibodies.

### Immunoprecipitation.

The plasmids were expressed via vaccinia virus T7 expression system. Briefly, BHK-21 cells were preinfected with vaccinia MVA-T7 (61) and then transfected with pYM-13 or pCI empty vector by using SuperFect (Qiagen, Hilden, Germany). The expressed proteins were labeled with Tran^35^S-Label (Hartmann Analytics, Göttingen, Germany), as described previously (62). The supernatant of the cell cultures (SN) was collected to determine the secreted proteins, and the cells were washed three times with PBS before the cells were lysed. Both cell lysate (CL) and supernatant samples were prepared under denaturing conditions. The produced proteins in SN/CL that reacted with a specific antiserum directed against the HA tag were precipitated. When indicated in the text, the precipitates were treated before electrophoresis with 1 μl of PNGase F (New England Biolabs) for 1 h at 37°C, as suggested by the supplier. The samples were separated by SDS-PAGE under reducing conditions, and labeled proteins were detected on imaging plates using a CR-35 Bio image plate scanner and AIDA image analyzer 5 software (both the equipment and the software were from Elysia-Raytest, Straubenhardt, Germany).

### Virus rescue.

RNA was generated by *in vitro* transcription using the RiboMax-T7 large-scale RNA production system (Promega). All plasmids were linearized beforehand using SmaI. MDBK cells were freshly seeded into a 10.0-cm diameter dish and left to grow overnight to 100 % confluence. On the day of electroporation, the cells were detached using trypsin, washed once with PBS, and counted, and 10^5^ cells per electroporation were resuspended in cold PBS and electroporated as described previously (62). The cells were resuspended in 5 ml of medium, a 50-μl portion was transferred to a 24-well plate for immunofluorescence staining after 24 h, and the remaining cells were seeded in 24-well plates for cultivation.

To recover progeny virus, lysates of transfected cells were prepared by three freeze-thaw cycles. Next, 10% of the lysate from a well (24 plate) was used for reinfection. Virus infection was detected via immunofluorescence of PFA-fixed cells permeabilized with 0.05% Triton X-100 using the primary antibody Code4 (60) specific for NS3 at 24 to 48 h postinfection. For RNA electroporation, the MDBK-B2 cells were seeded to 10-cm cell culture dishes 1 day before electroporation. The cells in a 10-cm plate were sufficient for three electroporation (EP) samples.

First, the cells were detached from the dishes by treatment with trypsin mixture and resuspended in ZB5 with 10% FCS. The cells were then pelleted by centrifugation for 10 min at 1,000 rpm, the supernatant was removed, and the cells were washed once with ZB5. The cell pellet was then taken up in 1.3 ml of cold (∼4°C) PBS, and 0.4 ml was used for each electroporation RNA sample. Next, 3 to 5 μl of RNA was mixed with 0.4 ml of cells, followed by electroporation for 1 s at 180 V and 980 mF. After a second pulse using the same settings, the electroporated cells were immediately rinsed from the cuvette with ZB5 and transferred to two 3.5-cm cell culture dishes. The cells were observed to document the eventual development of a cytopathogenic effect.

The replication of electroporated RNA was demonstrated by immunofluorescence and the formation of infectious particles by reinfection experiments. For reinfection, the transfected cells were lysed by three cycles of freezing/thawing, and then part of the lysate was added to new cells. The successful reinfection was tested by immunofluorescence with NS3 primary antibody at 48 h postinfection.
